# A small population of hypothalamic neurons govern fertility: the critical role of VAX1 in GnRH neuron development and fertility maintenance

**Published:** 2016-08-02

**Authors:** Hanne M. Hoffmann, Pamela L. Mellon

**Affiliations:** Department of Reproductive Medicine, Center for Reproductive Science and Medicine, University of California, San Diego, La Jolla, CA 92037, USA

**Keywords:** Inherited infertility, GnRH neuron, VAX1, homeodomain transcription factor, hypogonadism, development

## Abstract

Fertility depends on the correct maturation and function of approximately 800 gonadotropin-releasing hormone (GnRH) neurons in the brain. GnRH neurons are at the apex of the hypothalamic-pituitary-gonadal axis that regulates fertility. In adulthood, GnRH neurons are scattered throughout the anterior hypothalamic area and project to the median eminence, where GnRH is released into the portal vasculature to stimulate release of luteinizing hormone (LH) and follicle-stimulating hormone (FSH) from the pituitary. LH and FSH then regulate gonadal steroidogenesis and gametogenesis. Absence of GnRH neurons or inappropriate GnRH release leads to infertility. Despite the critical role of GnRH neurons in fertility, we still have a limited understanding of the genes responsible for proper GnRH neuron development and function in adulthood. GnRH neurons originate in the olfactory placode then migrate into the brain. Homeodomain transcription factors expressed within GnRH neurons or along their migratory path are candidate genes for inherited infertility. Using a combined *in vitro* and *in vivo* approach, we have identified Ventral Anterior Homeobox 1 (*Vax1*) as a novel homeodomain transcription factor responsible for GnRH neuron maturation and fertility. GnRH neuron counts in *Vax1* knock-out embryos revealed *Vax1* to be required for the presence of GnRH-expressing cells at embryonic day 17.5 (E17.5), but not at E13.5. To localize the effects of *Vax1* on fertility, we generated *Vax1^flox^* mice and crossed them with *Gnrh^cre^* mice to specifically delete *Vax1* within GnRH neurons. GnRH staining in *Vax1^flox/flox^:GnRH^cre^* mice show a total absence of GnRH expression in the adult. We performed lineage tracing in *Vax1^flox/flox^:GnRH^cre^:RosaLacZ* mice which proved GnRH neurons to be alive, but incapable of expressing GnRH. The absence of GnRH leads to delayed puberty, hypogonadism and complete infertility in both sexes. Finally, using the immortalized model GnRH neuron cell lines, GN11 and GT1-7, we show that VAX1 is a direct regulator of *Gnrh1* transcription by binding key ATTA sites within the *Gnrh1* promoter. This study identifies VAX1 as a key transcription factor regulating GnRH expression and establishes VAX1 as a novel candidate gene implicated in heritable infertility.

Infertility classified as idiopathic hypogonadotropic hypogonadism (IHH) is characterized by delayed or absent sexual maturation, and low gonadotropin and sex steroid levels due to hypothalamic-pituitary-gonadal (HPG) axis deficiency (Figure 1) ^[1, 2]^. Due to the complexity of fertility regulation by the HPG axis, most cases of inherited infertility still have unknown genetic origins (Figure 1). Most genetic mutations known to cause IHH are autosomal recessive or dominant, however, it is becoming increasingly clear that a number of the unidentified genetic causes of IHH result from mutations in at least two distinct genes (complex heterozygosity). Despite the difficulty in detecting polygenic IHH, haploinsufficiencies adversely affecting fertility have been reported in both rodents and humans ^[3–7]^.

Gonadotropin-releasing hormone (GnRH) neurons are localized at the apex of the HPG axis (Figure 1) and originate outside the brain in the olfactory placode. In the mouse, these GnRH neurons arise at embryonic day 10.5 (E10.5), then migrate through the cribriform plate, reaching their final destination in the anterior hypothalamic area between E15 to E18, when approximately 800 GnRH neurons are found in the brain. Abnormal GnRH neuron maturation, migration, or GnRH secretion results in failures of puberty, fertility, and reproductive function. GnRH neuron maturation is key in maintaining fertility. Thus, to identify novel genes important for GnRH neuron development, we compared gene expression levels in two immortalized GnRH cell lines: the immature, migratory GnRH cell line (GN11), and the mature, post migratory, GnRH secretory cell line (GT1-7) ^[8, 9]^. The migration of GnRH neurons is principally restricted to the ventral forebrain, where homeodomain transcription factors expressed ventrally between E10 and E18 are involved in the correct maturation and migration of these neurons ^[6, 10–12]^. Comparison of RNA sequencing data from GN11 and GT1-7 identified one such gene, Ventral anterior homeobox 1 (*Vax1*). *Vax1* is differentially expressed between GN11 and GT1-7, and presents with a developmental expression profile overlapping with the area and timing of GnRH neuron migration as determined by comparing *Vax1* and *Gnrh1* expression patterns in the developing mouse brain on www.brain-map.org. VAX1 is a homeodomain transcription factor critical for embryonic development and essential for the formation of the eye, ventral forebrain and palate ^[13–15]^. In the adult mouse, *Vax1* is expressed at all levels of the reproductive axis: GnRH neurons, the testis, and the pituitary, but is absent in the pituitary gonadotropes and ovaries ^[16]^. We first determined if *Vax1* was involved in GnRH neuron development. We collected *Vax1* wildtype, heterozygote and knock-out embryos at two developmental time points: E13.5, when most GnRH neurons are localized in the olfactory placode, and are starting to migrate toward the cribriform plate, and at E17.5, when most GnRH neurons have completed their migration to the hypothalamus. At E13.5, there were normal numbers of GnRH neurons in *Vax1* knock-out mice. In stark contrast, at E17.5, ~50% of GnRH neurons were detected in the *Vax1* heterozygote embryos, and none in the knock-out ^[17]^. Thus, VAX1 is not required for generation of GnRH neurons, but instead for their maturation. As *Vax1* knock-out is perinatal lethal ^[15]^, and we observed a dosage effect of *Vax1* on GnRH neuron numbers, we investigated the impact on fertility in *Vax1* heterozygote mice. In agreement with what was found in E17.5 *Vax1* heterozygote embryos, adult *Vax1* heterozygote mice of both sexes had approximately 60% fewer GnRH-expressing neurons than control littermates. A fertility study of *Vax1* heterozygote males and females determined that both sexes were subfertile, *Vax1* heterozygote females had smaller and fewer litters than controls, whereas *Vax1* heterozygote males fathered smaller litters. The subfertility of female *Vax1* heterozygote mice was associated with a slight increase in circulating LH and estrogen levels, which was accompanied by prolonged and irregular estrous cycles. However, as *Vax1* was not expressed in the ovary or the pituitary gonadotropes, the pituitary cell population releasing FSH and LH (Figure 1), we concluded that female subfertility originated at the level of the GnRH neuron ^[16]^. In contrast, the sub-fertility of the *Vax1* heterozygote male, which was caused by an 80% reduction in the motile sperm population, could not be fully accounted for by the reduction in GnRH neurons as these mice were capable of maintaining normal LH, FSH, and testosterone levels. This suggests a combined effect of *Vax1* in GnRH neuron development and an unknown role in the testis leading to sub-fertility in *Vax1* heterozygote males ^[16]^.

To determine the contribution of VAX1 to GnRH neurons specifically, we generated a *Vax1^flox^* mouse and crossed it with a *GnRH^cre^* mouse to generate a conditional knock-out *Vax1^GnRH-cre^* mouse ^[18]^. *Vax1^GnRH-cre^* mice appear healthy and are indistinguishable from control littermates. Remarkably, adult *Vax1^GnRH-cre^* mice have no GnRH-expressing cells as determined by GnRH immunohistochemistry, leading to extremely low circulating FSH and LH levels. In the female *Vax1^GnRH-cre^* mouse, this resulted in delayed vaginal opening, an external marker of pubertal onset, hypogonadism (Figure 2), absence of mature ovarian follicles, and complete infertility. The low LH and FSH levels, in combination with estrogen levels below assay detection limits, correlated with an incapacity of *Vax1^GnRH-cre^* mice to progress through the estrous cycle, as evaluated by vaginal smears, and resulted in females being in permanent diestrus. In line with this, male *Vax1^GnRH-cre^* mice also presented with low LH and FSH levels, two hormones required for pubertal onset and normal testicular function. Indeed, *Vax1^GnRH-cre^* males had delayed pubertal onset as determined by preputial separation, a micropenis, were hypogonadal (Figure 2) with immature testes which were azoospermic, leading to complete infertility. To confirm that this infertility was due to absence of GnRH expression, and not due to an incapacity of the pituitary to release LH in response to GnRH, we performed a GnRH challenge. Indeed, an *intra-peritoneal* (*ip*) injection of GnRH resulted in a fold increase of LH release in both male and female *Vax1^GnRH-cre^* mice comparable to controls. In contrast, *ip* injection of the GnRH neuron activator kisspeptin (Figure 1), only allowed increased LH release in controls, and not in *Vax1^GnRH-cre^* mice. This localizes the origin of infertility of *Vax1^GnRH-cre^* mice at the level of the GnRH neuron, and excludes a contribution of the pituitary in their infertility. Evaluation of heterozygote *Vax1^GnRH-cre^* (*Vax1^flox/+^:GnRH^cre^*) recapitulated most of the subfertility phenotype of the full body *Vax1* heterozygote mouse, but not all, indicating that *Vax1* has a role in fertility maintenance outside of the GnRH neuron. To determine the destiny of GnRH neurons in *Vax1^GnRH-cre^* mice, we performed lineage tracing of GnRH neurons using *Vax1^GnRH-cre^:RosaLacZ^+^* mice. This approach allows “Cre” to delete a Flox-Stop to activate LacZ ^[19]^ and thus marks all GnRH^cre^ expressing cells with LacZ permanently regardless of ongoing GnRH gene expression. The specific expression of LacZ in GnRH neurons allowed us to determine whether GnRH neurons were alive without expressing GnRH. In this scenario, LacZ staining would be detected, while GnRH staining would be absent. Lineage tracing showed comparable localization and numbers of LacZ expressing cells in both control and *Vax1^GnRH-cre^:RosaLacZ^+^* mice, proving that GnRH neurons in *Vax1^GnRH-cre^* mice stop expressing GnRH but survive. Thus, VAX1 is critical in maintaining GnRH expression after E13.5.

To determine if the effect of VAX1 on GnRH expression was direct, we next asked if VAX1 could directly regulate the *Gnrh1* promoter. To answer this, we used the two model GnRH cell lines, GN11 and GT1-7 cells. Transient transfections of GN11 and GT1-7 cells with various constructs of the *Gnrh1* promoter driving the expression of a luciferase reporter, allowed us to identify four conserved ATTA sites in the *Gnrh1* promoter potentially regulated by VAX1. To prove VAX1 directly interacted with the identified ATTA sites of the *Gnrh1* promoter, we performed electrophoretic mobility-shift assays to show direct DNA-protein interactions. Indeed, VAX1 was able to directly bind the identified ATTA sites of the *Gnrh1* promoter. In contrast to what we expected, our data suggested that, in GT1-7 cells, VAX1 was a repressor of *Gnrh1* transcription. To explain these findings, we hypothesized that VAX1 was a weak activator that could compete for binding to the identified ATTA sites with other homeodomain transcription factors that were stronger activators of *Gnrh1* transcription. One such transcription factor is SIX6 ^[10]^. First, we asked if VAX1 was able to act as an activator of an ATTA-multimer in GT1-7 cells, which indeed it was. Thus, VAX1 can increase transcription in the context of GT1-7 cells. As SIX6 is a strong activator of the *Gnrh1* promoter, replacing SIX6 with VAX1, a weak activator, would, in our experimental setting, show as a reduction in transcription levels. By cotransfecting various concentrations of VAX1 and SIX6 into GT1-7 cells, along with the *Gnrh1* promoter driving a luciferase reporter, we determined a complex competition between SIX6 and VAX1. Depending on the specific concentrations of these transcription factors, different levels of transcription were revealed. To our satisfaction, we found that VAX1 can compete with SIX6 for binding to the *Gnrh1* promoter, which to some extent can explain the absence of GnRH expression in *Vax1^GnRH-cre^* mice.

In summary, we have identified *Vax1* as a key transcription factor involved in maintaining GnRH expression after E13.5. Expression of GnRH is *Vax1* dose sensitive, and *Vax1* haploinsufficiency leads to subfertility. Thus, *Vax1* is a novel candidate gene for polygenic IHH. We show that the role of *Vax1* within the GnRH neuron is to maintain GnRH expression through a direct effect on the *Gnrh1* promoter. Absence of *Vax1* from GnRH neurons abolishes GnRH expression and leads to complete infertility and hypogonadism.

## Figures and Tables

**Figure 1:**
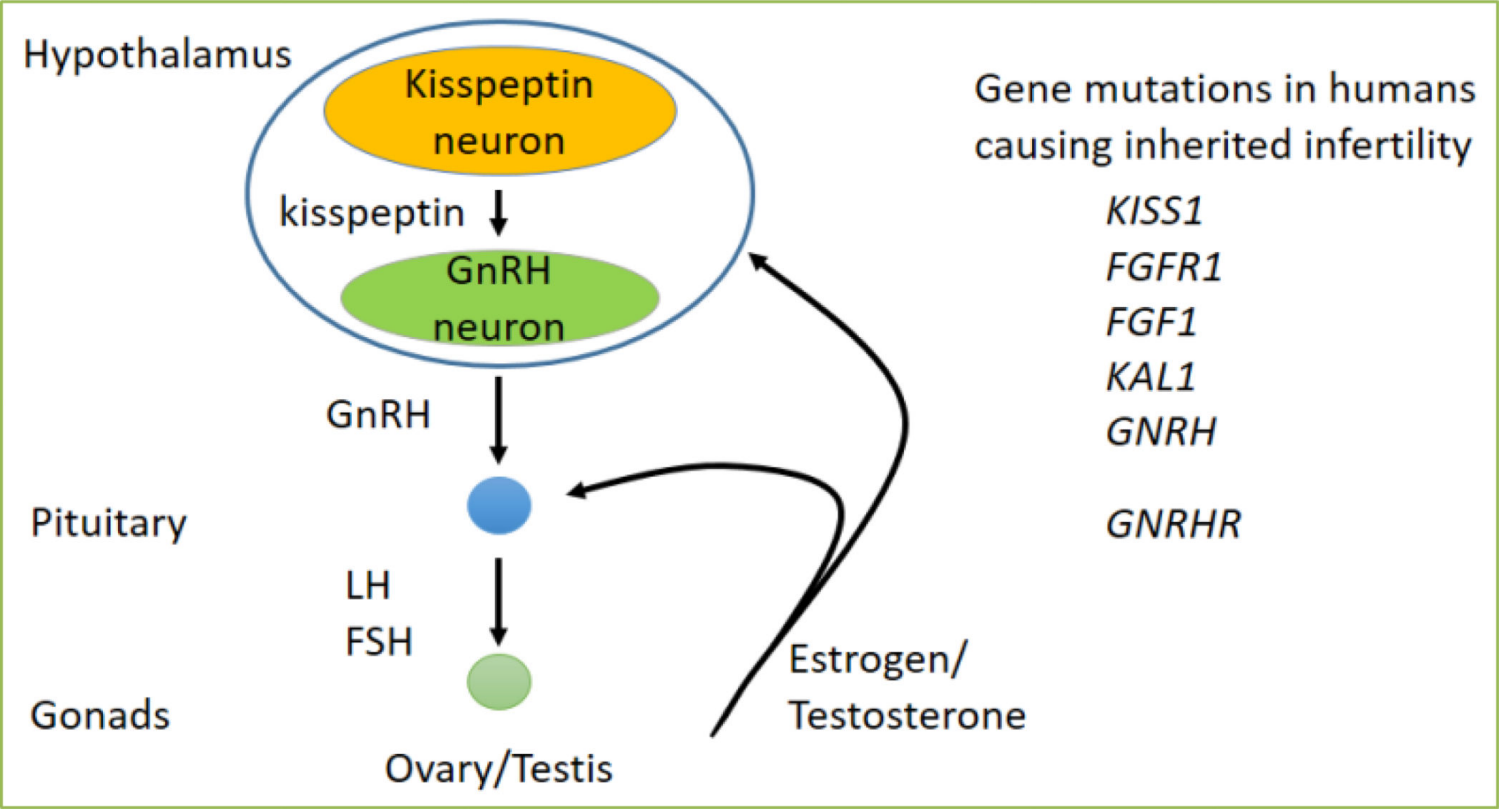
Mutations of genes in the hypothalamic-pituitary-gonadal axis cause inherited infertility The hypothalamic-pituitary-gonadal axis is controlled by kisspeptin input on to GnRH neurons. Pulsatile release of GnRH triggers LH and FSH release from the pituitary, which in turn stimulate the gonads to release sex steroids. Testosterone and estrogen (in the male and female, respectively), feedback to the hypothalamic kisspeptin neurons and gonadotropes in the pituitary. Mutations in key genes for GnRH or kisspeptin neuron function, or responsiveness of pituitary gonadotropes to GnRH, cause infertility.

**Figure 2:**
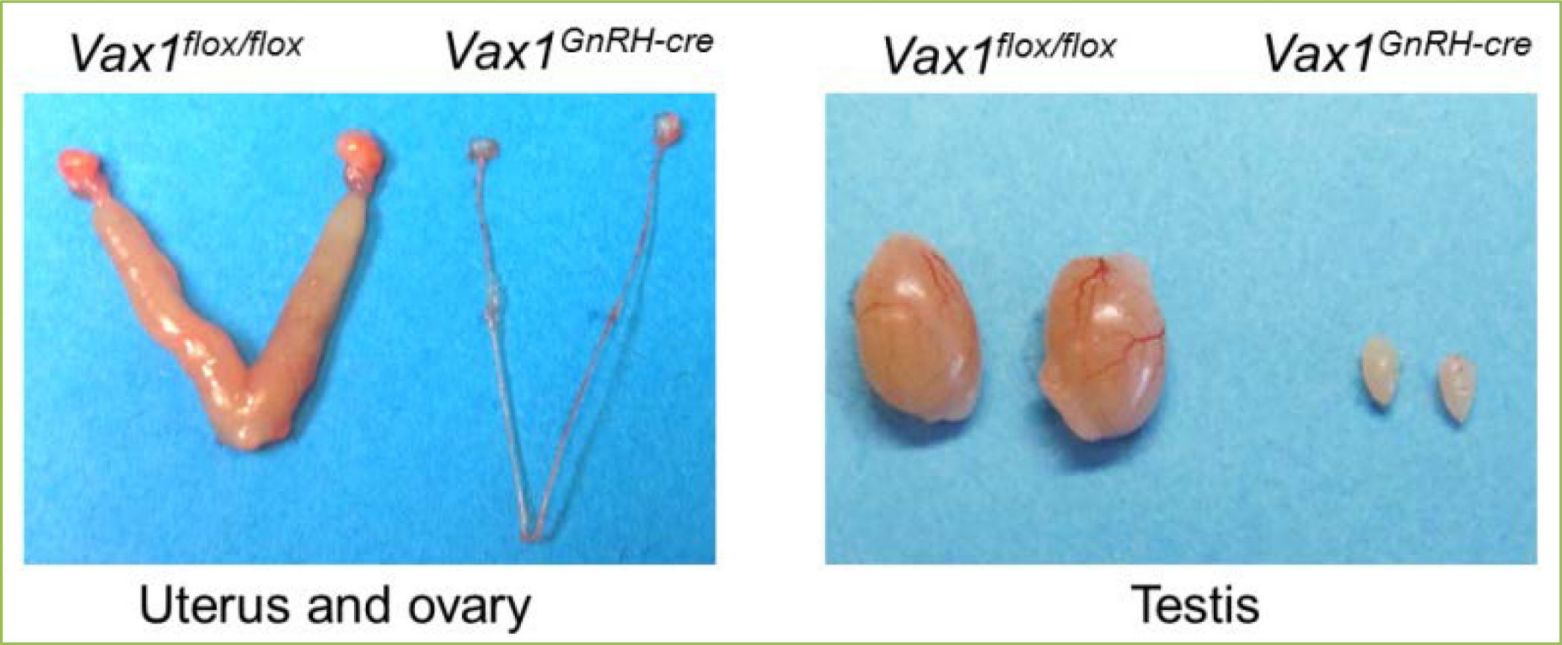
Deletion of *Vax1* from GnRH neurons leads to hypogonadism *Vax1^GnRH-cre^* mice have no GnRH expression, leading to female (left) and male (right) hypogonadism.

## Abbreviations

Vax1: Ventral Anterior Homeobox 1 gene
GnRH: Gonadotropin-Releasing Hormone
LH: luteinizing hormone
FSH: follicle-stimulating hormone
E: embryonic day
IHH: idiopathic hypogonadotropic hypogonadism
HPG: hypothalamic-pituitary-gonadal
ip: intra-peritoneal
